# EvaLuation of Interventions for informed Consent for randomIsed controlled Trials (ELICIT): developing a core outcome set

**DOI:** 10.1186/1745-6215-16-S1-P5

**Published:** 2015-05-29

**Authors:** Katie Gillies, Cynthia Fraser, Vikki Entwistle, Shaun Treweek, Paula Williamson, Marion Campbell

**Affiliations:** 1 Health Services Research Unit, University of Aberdeen, Aberdeen, AB25 2ZB, UK; 2 Department of Biostatistics, University of Liverpool, Liverpool, L69 3GS, UK

## Background

The process of obtaining informed consent for participation in randomised controlled trials (RCTs) was established as a mechanism to protect participants against undue harm from research and allow people to recognise any potential risks, or benefits, associated with the research. A number of interventions have been put forward to improve this process. Outcomes reported in trials of interventions to improve the informed consent process for decisions about trial participation tend to focus on ‘understanding’ of trial information. However, the operationalization of understanding as a concept, the tools used to measure it, and the timing of the measurements are heterogeneous. The measurement tools are often study-specific non-validated tools with only a handful of validated tools being implemented. Moreover, there is a lack of clarity regarding which outcomes matter (to whom) and why. This inconsistency between studies results in difficulties when making comparisons across studies as evidenced in two recent systematic reviews of informed consent interventions. As such, no optimal method for measuring the impact of these interventions aimed at improving informed consent for RCTs has been identified.

The ELICIT project aims to develop a core outcome set for the evaluation of interventions intended to improve informed consent for RCTs.

## Methods

The project will adopt and adapt methodology previously developed and used in projects developing core outcome sets for assessment of clinical treatments. Specifically, the work will consist of three stages: 1. A systematic methodology review of existing outcome measures of trial informed consent interventions; 2. Interviews with key stakeholders to explore additional outcomes relevant for trial participation decisions; and 3. A Delphi study to refine the core outcome set for evaluation of trial informed consent interventions.

## Conclusion

This presentation will discuss the key issues relevant for this work and present data generated to date from the systematic literature review, which is reviewing both quantitative and qualitative evidence.

